# Helminth parasites and immune regulation

**DOI:** 10.12688/f1000research.15596.1

**Published:** 2018-10-23

**Authors:** Pedro H. Gazzinelli-Guimaraes, Thomas B. Nutman

**Affiliations:** 1 Laboratory of Parasitic Diseases, National Institute of Allergy and Infectious Diseases, National Institutes of Health, 4 Center Drive, Building 4, Room 211, Bethesda, MD, 20892, USA

**Keywords:** helminth, immune regulation, parasites, immune response, type-2 immunity, regulatory response.

## Abstract

Helminth parasites are complex metazoans that belong to different taxonomic families but that collectively share the capacity to downregulate the host immune response directed toward themselves (parasite-specific immunoregulation). During long-standing chronic infection, these helminths appear able to suppress immune responses to bystander pathogens/antigens and atopic, autoimmune, and metabolic disorders. Helminth-induced immunoregulation occurs through the induction of regulatory T cells or Th2-type cells (or both). However, secreted or excreted parasite metabolites, proteins, or extracellular vesicles (or a combination of these) may also directly induce signaling pathways in host cells. Therefore, the focus of this review will be to highlight recent advances in understanding the immune responses to helminth infection, emphasizing the strategies/molecules and some of the mechanisms used by helminth parasites to modulate the immune response of their hosts.

## Introduction

Helminth parasites belong to a diverse group of complex metazoans from different taxonomic families. Collectively, helminth infections are a major public health problem worldwide, and recent estimates suggest that 1.5 billion people have one or more of the common helminth infections ( Table 1), most of whom reside in low- and middle-income countries in the endemic areas of Asia, Latin America, the Caribbean, and sub-Saharan Africa ^1^.

**Table 1.** Human helminth infections of public health importance.

Helminth species	Disease or condition in humans	Estimate prevalence worldwide	Habitat of adult worm in humans
Nematodes			
*Ascaris lumbricoides*	Ascariasis	804 million	Small intestine
*Ascaris suum*			
*Trichuris trichiura*	Trichuriasis	477 million	Large intestine
*Enterobius vermicularis*	Enterobiasis (Oxyuriasis)	>200 million	
*Toxocara canis*	Visceral or ocular larva migrans	Unknown	N/A
*Necator americanus*	Necatoriasis	472 million	Small intestine
*Ancylostoma duodenale*	Ancylostomiasis		
*Ancylostoma ceylanicum*			
*Strongyloides stercoralis*	Strongyloidiasis	30–100 million	
*Wuchereria bancrofti*	Lymphatic filariasis	44 million	Lymphatic vessels
*Brugia malayi or Brugia timori*			
*Onchocerca volvulus*	Onchocerciasis (river blindness)	17 million	Subcutaneous tissue
*Trichinella spiralis*	Trichinellosis	0.066 million	Small intestine
Trematodes			
*Schistosoma mansoni*	Intestinal schistosomiasis	206 million	Mesenteric veins
*Schistosoma haematobium*	Urogenital schistosomiasis		Venous plexus of urinary bladder
*Schistosoma japonicum*	Intestinal schistosomiasis		Mesenteric veins
*Fasciola hepatica*	Fascioliasis	80 million	Bile ducts
*Clonorchis sinensis*	Clonorchiasis		Bile ducts and gall bladder
*Opisthorchis spp.*	Opisthorchiasis		
*Paragonimus spp.*	Paragonimiasis		Lungs
Cestodes			
*Echinococcus granulosus*	Hydatid disease	0.8 million	N/A
*Echinococcus multilocularis*	Alveolar echinococcosis	0.019 million	N/A
*Cysticercus cellulosae* ( *Taenia solium* larva)	Cysticercosis and Neurocysticercosis	1 million	N/A
*Taenia solium*	Intestinal taeniasis	0.38 million	Small intestine

N/A, not applicable. There is no development of adult worms in humans.

These many helminths each have significant differences in their biological life cycles along with marked variation in tissue tropism. These differences are reflected in the differences in clinical outcomes seen among the helminth parasites. Pathologic consequences of most helminth infection have been associated with both the parasite intensity (or burden) and the relative acuteness or chronicity of the infection.

Despite these helminth species-specific differences, helminths as a group have been shown to modulate/regulate the host response to themselves (parasite-specific immunoregulation) ^2– 4^. However, with long-standing chronic infection, these parasites can alter the immune response to bystander pathogens/antigens ^5, 6^, including vaccines ^7, 8^, and allergens ^9, 10^. In addition, they have been associated with modulation of the severity of inflammatory bowel disease (IBD) ^11^, diabetes ^12^, and arthritis ^13^.

Because of the helminths’ capacity to regulate the host immune response, a regulation that can be partially reversed by anthelmintic therapy, there has been widespread interest in understanding the mechanisms underlying helminth-induced immune regulation along with those parasite-encoded molecules that may be driving such regulation. In particular, the so-called excretory/secretory (ES) products from helminth parasites have gained the most attention, as they may be targets for anthelmintic vaccines, diagnostics, and drugs or they could be useful as potential therapeutics for inflammatory and autoimmune disorders. Therefore, the focus of this review will be to highlight recent advances in understanding the immune responses to helminth infection, emphasizing the strategies/molecules used by helminth parasites to modulate the immune response of their hosts.

## Acuteness and chronicity of infection drive distinct immune profiles

The complexity of the life cycles of helminth parasites that have multiple developmental stages of the parasite each with a distinct antigenic repertoire and often distinct tropisms for particular organ systems (for example, intestinal and airway mucosa in larval *Ascaris lumbricoides* and hookworm infections; skin/subcutaneous tissue and draining lymph nodes in *Onchocerca volvulus* infection; the hepatic portal system for *Schistosoma mansoni*; and the muscle and the brain for *Taenia solium* cysticerci) makes it difficult to generalize about helminths as a single group ^2^. Normally, however, infection occurs through the ingestion of eggs or exposure to infective larvae. Once in contact with their mammalian hosts, the parasite progressively develops during the migration of the larval stages through the host’s systems/organs that culminate in their maturation into adult worms within a specific habitat that reflects each helminth’s tropism for a particular anatomical niche. As these developmental transitions and migration occur over a period of time (from weeks to years, depending on the parasite and its particular mammalian host), immune responses are often regulated differently on the basis of the resident tissue or perhaps by the life span of the parasite.

One example of the complex developmental and migratory processes that occur following helminth infection is that caused by the roundworms *A. lumbricoides*, a parasite that, by current estimates, is harbored by more than 800 million people worldwide ^14^. Human infection occurs following the ingestion of parasite eggs containing the third-stage infective larvae (L3) that hatch in the small intestine. After penetrating the intestine at the level of the caecum or proximal colon, these L3 migrate through the portal vessels to the liver and subsequently to the lungs. There they migrate through the lung parenchyma and penetrate into the alveolar spaces, causing a range of symptoms, including wheezing, dyspnea, cough, and substernal pain ^15, 16^. This early/acute phase of infection has been called larval ascariasis ^17^. These migrating *Ascaris* larvae induce a local inflammatory response in the lungs of humans (causing a Löffler’s-like syndrome ^18^) and of experimentally infected mice. In mice, the inflammation has been characterized as a type 2 response (dominated by IL-4 and IL-13 and some IL-5). Tumor necrosis factor-alpha (TNF-α) and interleukin-1 beta (IL-1β) levels are also seen in the lung induced by larval migration. At the peak of *Ascaris* larval migration (~8 days post-infection), there is a marked production of IL-6, thought to be related to the prominent neutrophil infiltration ^19^. When the larvae start to leave the lung tissue to migrate back to the small intestine to complete their life cycle, the neutrophil infiltrate in the lung is replaced by both alternatively activated (or M2) macrophages (AAM) (Fizz1+, Arginase-I+) and eosinophils that play a key role in tissue remodeling and prevention of re-infection ^20^. Once back in the small intestine, the larvae mature into adult worms, establishing a long-term chronic infection characterized by a profoundly diminished helminth-specific response ^21, 22^.

Over the last 20 years, several experimental studies using intestinal nematodes of rodents such as *Heligmosomoides polygyrus* or *Nippostrongylus brasiliensis* have provided a detailed description of a “protective” immune response associated with worm expulsion ^23– 26^. Although the mechanisms of larval killing are less well-studied, it is known that early in infection, prior to adult worm development and establishment, mucosal epithelial sensor cells secrete a group of alarmins—for example, IL-25, thymic stromal lymphopoietin (TSLP), and IL-33—that promote the activation and differentiation of innate and adaptive type 2 cells, leading to the secretion of a myriad of cytokines, including IL-4, IL-5, IL-9, and IL-13 ^26, 27^. These type 2-associated cytokines result in goblet cell hyperplasia, mucus hyper-secretion, and smooth muscle contraction and other immunological changes such as eosinophilia and the differentiation of AAM macrophages ^26, 28^.

Recently, a novel subset of epithelial cells, termed tuft cells, was identified in the small intestine. These tuft cells constitutively express IL-25. Von Moltke *et al*. ^29^ and Gerbe *et al*. ^30^ showed that after infection by the rodent hookworm *N. brasiliensis*, tuft cells produce IL-25 that in turn activates type 2 innate lymphoid cells (ILC2s) to produce IL-13 that subsequently acts on epithelial crypt progenitors to promote differentiation and increased frequency of both tuft and goblet cells. As reviewed by Grencis and Worthington ^31^, this tuft cell–ILC2 circuit loop orchestrates a rapid and effective anti-helminth immune effector response that leads to worm expulsion.

For helminth infection in humans, the immune response during the early/acute phase of infection involves the induction of type 2-associated cytokines (IL-4, IL-5, IL-9, and IL-13) first by innate lymphocytes (ILC2) and later by effector antigen-specific polyfunctional CD4 T cells ^32^. This relatively early phase also induces high antigen-specific IgG4 and IgE levels as well as peripheral and tissue eosinophilia and expanded populations of AAM ^33, 34^.

In peripheral blood, this polarized type 2 response occurs at the time of patency when egg laying (for example, *S. mansoni*) ^35^ or microfilarial release (for example, *Wuchereria bancrofti*) from adult females occurs ^36^, resulting in a significant modulation of Th1 responses (IL-2 and interferon-gamma [IFN-γ]). However, this persistent dominant Th2 response over the course of the helminth infection also induces expansion of natural ^37– 39^ and helminth-induced ^40, 41^ regulatory T (Treg) cells and immunoregulatory monocytes ^42– 44^; this same response drives B-cell class-switching to IgG4 ^45^. This new regulatory environment, characterized by low parasite antigen-specific lymphocyte proliferation, higher antigen-specific IgG4/IgE ratios, and increased levels of the regulatory cytokines IL-10 and transforming growth factor-beta (TGF-β), is the hallmark of an asymptomatic, chronic infection ^46– 49^.

In chronic filarial infections, microfilaremia is observed in clinically asymptomatic patients. Interestingly, T cells from these filarial-infected asymptomatic patients show the following: a muted/anergic parasite-specific lymphoproliferative response ^50– 52^; an increased parasite-specific IL-4/IFN-γ ratio ^46^; dysfunctional antigen-presenting cells (APCs) ^53, 54^; expanded natural Treg (nTreg) cells expressing CTLA-4, PD-1, and GITR (molecules associated with regulatory functions on nTreg cells) ^55^; and elevated IL-10 levels ^48, 56^. In contrast, infected patients with progressive and often symptomatic infection, such as elephantiasis, fail to suppress (or be tolerant to) filarial antigen-driven inflammation. This relative immune hyper-responsiveness is associated with microfilarial clearance but also consequent morbidity ^56^. Furthermore, anthelmintic therapy that leads to clearance of the microfilariae or *in vitro* blockade of IL-10 can result in a recovery of many of the parasite antigen-specific responses, suggesting that they were actively inhibited in the presence of the parasites or of circulating parasite antigens ^56, 57^.

**Figure 1.**
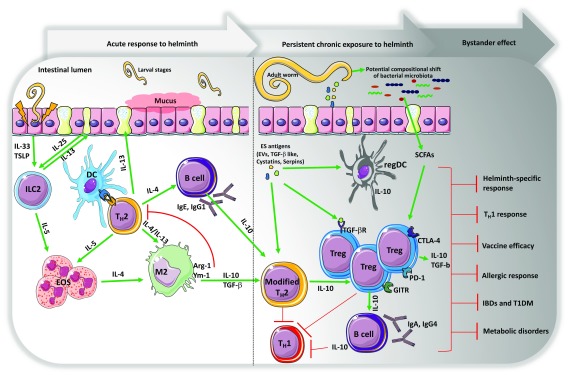
Acuteness and chronicity of helminth infection drive distinct immune profiles. Early in infection, normally during the larval migration through the lungs or intestinal mucosa, prior to adult worm development and establishment, epithelial cells secrete a group of alarmins—thymic stromal lymphopoietin (TSLP) and interleukin-33 (IL-33), including IL-25-producing tuft cells—that promote the activation and differentiation of type 2 innate lymphoid cells (ILC2) and polyfunctional CD4 T helper 2 (Th2) cells, leading to the secretion of a myriad of cytokines, including IL-4, IL-5, and IL-13. These type 2-associated cytokines result in goblet cell hyperplasia, mucus hyper-secretion, peripheral and tissue eosinophilia, and differentiation of M2 macrophages and also induce high antigen-specific IgG1 and IgE levels. Helminth early/acute responses generally associate with an allergy-like response. The persistent exposure to helminth parasites and helminth-derived excretory/secretory (ES) antigens over the course of the infection lead to a modified type 2 response resulting in a significant modulation of T helper 1 (Th1) response—IL-2 and interferon-gamma (IFN-γ)—and also induce the expansion of natural regulatory T (nTreg) cells expressing CTLA-4, PD-1, GITR, and regulatory dendritic cells (regDCs) and monocytes, which are all sources of IL-10. This same response drives B-cell class-switching to IgG4. Chronic infection with helminth also alters the composition of intestinal bacterial communities leading to more microbial-derived short chain fatty acids (SCFAs) that also activate and promote the expansion of Treg cells. Collectively, this new regulatory environment is the signature for the establishment of an asymptomatic chronic long-standing infection, characterized by a muted/anergic parasite-specific lymphoproliferative response but also a suppressed immunity to bystander pathogens, allergens, vaccines, or non-related inflammatory, autoimmune—inflammatory bowel diseases (IBDs) and type 1 diabetes (T1DM)—or metabolic diseases. DC, dendritic cell; EOS, eosinophil; EV, extracellular vesicle; TGF-β, transforming growth factor beta.

Traditionally, it has been shown that, beyond attenuating parasite-specific response, helminths can suppress the immunity to bystander pathogens or to vaccines ^7, 58^. It is known that the induction of the regulatory response by helminths is associated with the downmodulation of Th1 response ^3, 59, 60^, considered crucial for the immunological control of viral, bacterial, or protozoal infections ( Figure 1). Immuno-epidemiological studies suggest that coincident infection with helminths has a strong potential to significantly influence the course of viral or protozoan infections, especially in those infections where protective immunity depends on a strong Th1/Th17 immune response ^61– 63^. In addition, several recent studies have provided insight into how helminths and helminth-derived molecules (ES products) regulate some of the inflammatory responses that underlie allergic, autoimmune, or metabolic disorders.

## Helminth-derived excretory/secretory products: the era of the extracellular vesicles

Helminth-induced immune responses have long been postulated to be directed at the ES products from living parasite stages during the infection. Some of the soluble proteins, lipids, and carbohydrates present in the ES products have been shown to have immunomodulatory activity ^64, 65^. The list of helminth-derived immunomodulatory molecules that evoke a regulatory phenotype among innate and adaptive immune cells has been increasing over the last decade ^9, 10, 41, 64– 66^.

The relatively recent discovery of extracellular vesicles (EVs) secreted by helminths has suggested a new paradigm in the study of host–parasite interaction ^67, 68^. EVs are released from most cell types and from a diverse group of pathogens, including parasitic helminths ^69, 70^. At homeostasis, EVs represent a mechanism by which cell-to-cell communication occurs through the transfer of genetic material, proteins, and lipids ^68^. In parasitic infections, EVs can function by transmitting signals between parasites, from parasite to host cells, or from the host to the environment ^68^.

In general, it is felt that helminth EVs have immunoregulatory effects on host cells ^71, 72^. For a group of helminths, the analysis of the composition of these EVs has identified proteins previously described in ES products along with microRNAs (miRNAs), a highly conserved group of small, non-coding RNA molecules that can control gene expression. Among the proteins identified as components of helminth EVs are cysteine protease inhibitors (cystatins), serine protease inhibitors (serpins), metabolic enzymes such as enolase, GAPDH, and aldolase, and the well-known exosome components Hsp70, Hsp90, and annexins ^73^.

Recently, it has been shown that EVs secreted by both the parasite and the host can influence the outcome of an infection. With an experimental murine model for a chronic helminth infection ( *H. polygyrus*), it was shown that EVs secreted by the *H. polygyru*s are internalized by murine macrophages and, as a consequence of this internalization, suppress the activation of both M1 and M2 macrophages ^72^. In contrast, with the infective stage of the filarial parasite *Brugia malayi*, it has been shown that these parasites secrete EVs containing parasite protein and miRNAs, which are also internalized by macrophages but which elicit/induce macrophage (M1) activation ^74^. Finally, with *H. polygyru*s and rodent filarial nematode *Litomosoides sigmodontis*, it was shown that these parasites secrete EVs containing miRNAs, which when administered prior to allergic sensitization in an experimental allergy-asthma model in mice actually suppressed the allergen-induced type 2 innate immune response *in vivo* ^71^.

Notwithstanding the data demonstrating EV-induced suppression of host inflammation and immune response, some groups have advocated the use of helminth-derived EVs for the identification of targets to be used in vaccines against some helminth infections ^69, 73, 75^. Indeed, EVs isolated from the ES products of *Trichuris muris* (a whipworm of mice) can induce protective immunity, reducing about 60% of parasite burden, in a murine model when administered as a vaccine without adjuvant, generating a strong EV-specific antibody response ^76^. Moreover, helminth-derived EVs induced protection to *H. polygyrus* larval challenge in mice ^72^.

Interestingly, there has been a suggestion that helminth-derived EVs could be used as therapeutics to regulate inflammation in the context of allergic, autoimmune, and metabolic disorders ^71, 77, 78^. As suggested by Siles-Lucas *et al*. ^78^, specific molecules from helminth exosomes could be delivered in artificial exosomes to host cells with the aim of regulating pathologic inflammatory responses. How to target specific cells, to stabilize these EVs, and to find the correct dosage are challenges that will need to be addressed.

## Allergic diseases and helminth infection

Allergies are inflammatory disorders that result generally from inappropriate immune responses to environmental allergens. Allergic sensitization or atopy is driven by allergen-specific responses initiated by CD4 ^+^ Th2 cells that ultimately drive the production of allergen-specific IgE ^79^. Although the hygiene hypothesis suggests that the lack of exposure in children early in their development to helminth parasites or other microbial products (as seen in high- and middle-income countries) may drive the increased incidence of allergic diseases seen in these countries, there are conflicting sets of studies in humans and in experimental models ^80– 83^ that have called this particular hypothesis into question. Leonardi-Bee *et al*. ^84^ demonstrated, in a meta-analysis, that chronic infection by the hookworm *Necator americanus* protects against asthma but that *A. lumbricoides* infection aggravates the clinical symptoms of this allergic condition. Interestingly, children living in a helminth-endemic region of Ecuador had a lower risk of allergies when compared with non-parasitized children in the same region ^85^. Moreover, repetitive anthelmintic treatment in endemic areas has been shown to increase the prevalence of allergen skin test reactivity in children ^86^.

The differences among these studies likely reflect differences in the timing of parasite infection in relationship to immune maturation or sensitization, although the species of the helminth, the intensity of the helminth infection, or the nature of the allergic disease assessed (or a combination of these) may also play a role in driving the outcomes seen. The most compelling explanation relates to the relative acuteness of the helminth parasitic infection, with early exposure to helminths driving an enhanced allergic inflammatory response ^32^ and long-term chronic infections attenuating the host allergic response ^58^.

Among the various hypotheses put forward to explain the modulatory influence of helminth infection on allergic effector responses in humans and murine models, the IL-10-induced suppression of Th2-effector responses and the expansion of natural and parasite-induced Treg cells ^9, 87, 88^ have been the leading candidates. One possible mechanism is the IL-10-induced inhibition of IgE signaling (key players in allergic diseases) in basophils ^89, 90^. Over the last decade, it has been shown that, in human parasitic infection and in experimental models of helminth infection, helminth parasites can induce B cells to differentiate into IL-10-producing regulatory B cells that may play a role in the suppression of the immune response that leads to an expansion of Treg cells ^91, 92^.

Other studies have suggested that helminths potentiate the functional effect of Treg cells by the secretion of parasite-derived TGF-β mimics. Helminth-derived TGF-β-like molecules can bind to TGF-β receptors and trigger FoxP3 ^+^ Treg cell expansion ^93– 96^. These data notwithstanding, new data suggest (based on *H. polygyrus* infection in mice) that the suppression of the type 2 allergic immune response in helminths is driven by a Hp-secreted protein (HpARI) that actively inhibits IL-33 release, thereby inhibiting the allergic response ^97^.

As reviewed recently, the ability of helminths to induce parasite-reactive Treg cells and IL-10 production may occur through parasite ES products ^64^. In addition, these helminth-derived products likely modulate bystander inflammatory responses, particularly the development of allergy ^9, 10, 64^. The molecular basis of this suppression has yet to be defined.

Recently, a novel mechanism underlying the helminth suppression of the allergic response has been suggested that implicates an interaction between helminth-derived proteins and the local microbiome ^98^. This concept stems from the “barrier regulation hypothesis of allergy” whereby, in the healthy state, a microbiome replete with mucosa-associated taxa stimulates the intestinal mucosa (mediated by IL-22) to produce a protective mucous layer and to produce anti-microbial peptides ^99^ that, in turn, regulate the abundance of particular bacterial communities. These bacteria-induced barrier-protective functions reduce the ability of allergens to cross the epithelial barrier ^99^. Compositional shifts within bacterial communities through dietary changes or antibiotic use can induce alterations in these bacteria-induced barrier-protective responses, thereby driving allergen-induced ILC2- or Th2-associated inflammation or both ^100^. A slight variation on this theme suggests that in an environment with chronic microbial exposure, the lung and gut microbiome stimulates the formation of regulatory dendritic cells that promote the differentiation of allergy-specific Treg cells that suppress allergen-induced Th2-associated inflammation ^79^.

Whether it is the helminth infection *per se* or helminth-derived proteins, changes in microbial composition/abundance/diversity appear to contribute indirectly to the modulation of the allergic response in the host ^100^. Indeed, it has been shown that chronic infection with *H. polygyrus* altered the intestinal bacterial communities ^101^ and, in so doing, increased the amount of microbial-derived short chain fatty acids (SCFAs) that in turn suppressed house dust mite-induced allergic inflammation ^98^.

## Helminth infections and autoimmune and metabolic disorders

Epidemiologic evidence demonstrates that while the prevalence of helminth infections is declining worldwide, the prevalence of autoimmune diseases—including IBDs and type 1 diabetes (T1DM)—and metabolic disorders is increasing rapidly. This phenomenon has led many to infer that there is a relationship between exposure to helminth infection and protection from autoimmune diseases—for example, Crohn’s disease (CD), ulcerative colitis (UC), and multiple sclerosis—and metabolic disorders. But how helminths regulate the group of varied inflammatory disorders, autoimmune diseases, and metabolic disorders remains unknown.

Using experimental model approaches, many authors have shown that helminth infection itself or treatment with helminth ES products is sufficient to suppress inflammation in numerous models of inflammatory diseases, including the dextran sodium sulfate (DSS)-induced colitis model in mice. ES products of *Ancylostoma ceylanicum* (human, cat, dog, and rodent hookworm) ^102^, *A. caninum* (dog hookworm) ^103^, *Trichinella spiralis* (carnivorous animal roundworm) ^104^, and *S. japonicum* (human blood fluke) have each been shown to attenuate the severity of DSS-induced colitis in mice ^105^. In addition, EVs of *N. brasiliensis* and *T. muris* ^77^ and the recombinant *B. malayi* protein rBmALT2 and cystatin ^106, 107^ have been shown to modulate colitis in experimental animal models. A common aspect of all of these studies has been the presence of increased levels of Th2-associated and regulatory cytokines (IL-10 and TGF-β) and a concomitant reduction in the inflammatory cytokines IL-6, IL-1β, IFN-γ, and IL-17a, known to be associated with the colitis-induced pathology. Concomitantly, two major species of helminths have been tested in more than 10 placebo-controlled clinical trials that have looked at *Trichuris suis* ova for the treatment of active UC and CD ^108^ or infection with *N. americanus* for the treatment of celiac disease in humans ^11, 109, 110^. As recently reviewed by Smallwood *et al*. ^111^, the results of the clinicals trials in humans are still controversial depending on the nature of the IBD or parasite evaluated, but, for some of them, there was some clinical improvement ^108, 109, 112^.

It has been shown that helminth infection can prevent T1DM based on the non-obese diabetic (NOD) mouse model. The data suggest that the immune switch from a Th1 to either a Th2 or a regulatory response is the primary mechanism through which T1DM is ameliorated ^12, 113^. In addition, it has been shown that helminth-derived proteins inhibit the initiation of autoreactive T-cell responses and prevent diabetes in the NOD mouse model ^114^. Interestingly, it has been postulated that the presence of these type 2 or regulatory cells in the pancreas of NOD mice has to take place before the bulk of beta cell mass is compromised by autoimmune attack ^115^. With a filarial infection in IL-4-deficient NOD mice, it was demonstrated that, despite the absence of a type 2 immune shift, filarial infection in IL-4-deficient NOD mice prevented the onset of T1DM and was accompanied by increases in CD4 ^+^CD25 ^+^Foxp3 ^+^ Treg cells ^40^. Moreover, blocking TGF-β signaling prevented the beneficial effect of helminth infection on T1DM, suggesting that skewing the immune response to a Th2 and regulatory environment could elicit suppression of the diabetogenic Th1 response.

Finally, when investigators evaluated the beneficial impact of helminth on protecting against the development of metabolic disorders, including obesity and dyslipidemia, commonly associated with insulin resistance and type 2 diabetes, parasite‐induced IL‐10 and the type 2 immune responses seem to act to improve insulin sensitivity ^116^, thereby ameliorating the metabolic syndrome (MetS)-associated morbidity ^117^. In this context, it has been shown that helminths have an important beneficial role by skewing this inflammatory response toward one with IL-4-producing eosinophils, M2 macrophages, and Treg cells that maintain insulin signaling and sensitivity ^118^.

## Future directions

Helminths are potent regulators of type 1 immune response induced by bystander pathogens or inflammatory disorders or both. Understanding the mechanisms underlying this interaction and identifying the potential molecular targets are the current challenges and areas that need to be investigated further to develop novel strategies to prevent or delay allergic, inflammatory, autoimmune, or metabolic disorders in humans.

## Abbreviations

AAM, alternatively activated macrophages; CD, Crohn’s disease; DSS, dextran sodium sulfate; ES, excretory/secretory; EV, extracellular vesicle; IBD, inflammatory bowel disease; IFN-γ, interferon-gamma; IL, interleukin; ILC2, innate lymphoid cell type 2; miRNA, microRNA; NOD, non-obese diabetic; nTreg, natural regulatory T; T1DM, type 1 diabetes; TGF-β, transforming growth factor-beta; Treg, regulatory T; UC, ulcerative colitis
